# Transcatheter Aortic Valve-in-Valve Implantation Complicated by Aorto-Right Ventricular Fistula

**DOI:** 10.1016/j.jaccas.2019.11.086

**Published:** 2020-02

**Authors:** Tiffany Patterson, Ronak Rajani, Giulia Esposito, Christopher Allen, Heath Adams, Bernard Prendergast, Christopher Young, Simon Redwood

**Affiliations:** a Cardiovascular Division, The Rayne Institute British Heart Foundation Centre of Research Excellence, King's College London, St. Thomas’ Hospital, London, United Kingdom; b Cardiothoracic Department, Guy’s and St. Thomas’ National Health Service Foundation Trust, London, United Kingdom

**Keywords:** fistula, sutureless bioprosthesis, TAVR, valve-in-valve, AVR, aortic valve replacement, BPV, bioprosthetic heart valve, CT, computed tomography, TEE, transesophageal echocardiography, THV, transcatheter heart valve, TTE, transthoracic echocardiography

## Abstract

We describe the case of a degenerative, sutureless bioprosthetic valve (BPV) with deformation and stent infolding in a patient with elevated surgical risk. Following discussion among the heart team, balloon valve fracture was performed to facilitate deployment of an aortic valve-in-valve transcatheter heart valve. Post-procedural imaging demonstrated BPV frame protrusion and contained annular rupture, which required operative intervention. (**Level of Difficulty: Intermediate.**)

## Central Illustration

**Figure:**
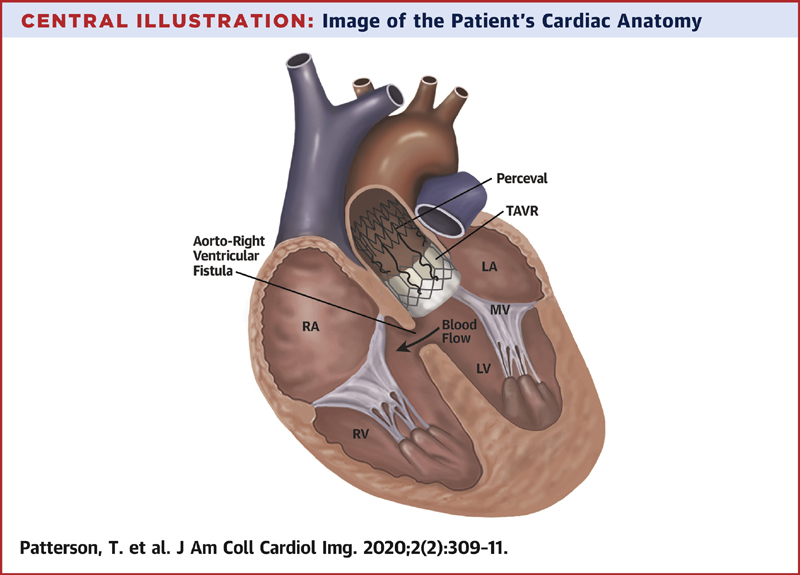

## Teaser

This paper describes the case of a degenerative, sutureless bioprosthetic valve (BPV) with deformation and stent infolding in a patient with elevated…

**Figure 1:**
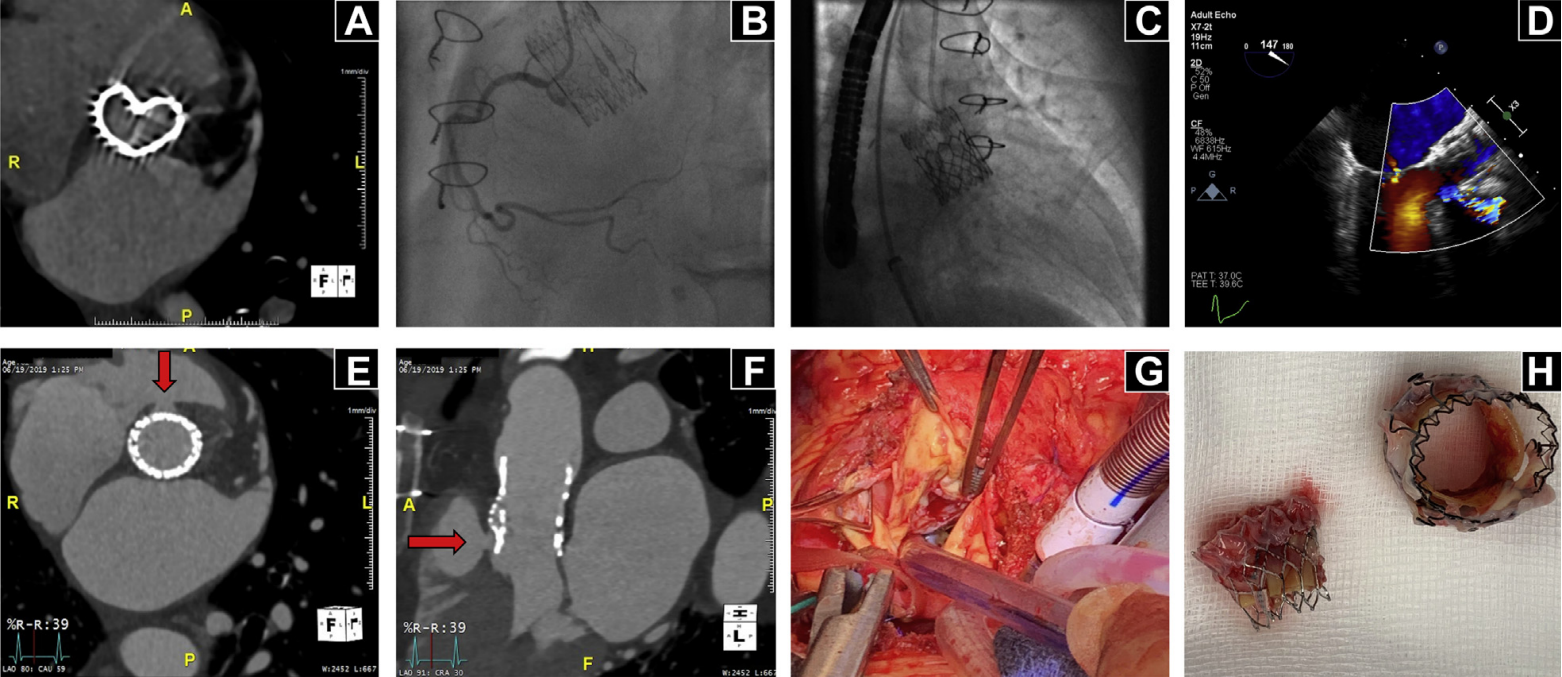
CT Demonstrates Stent Infolding, Distortion, and Collapse of Perceval Sutureless Valve **(A)** CT imaging demonstrates stent infolding, distortion, and collapse of Perceval Sutureless valve. **(B)** Left anterior oblique view of a Perceval Sutureless valve during right coronary angiography. **(C)** Orthogonal view of a successful Sapien S3 valve-in-valve implant. **(D)** Mid-esophageal long-axis view on TEE demonstrates a fistulous connection (white arrow shows color Doppler flow) between the right coronary cusp and right ventricle. Axial **(E)** CT imaging of well-deployed valve-in-valve with fistulous connection **(white arrow)** confirmed by contrast leak **(F)**. This is also demonstrated by 3-dimensional CT reconstruction (white arrow). **(G)** Resternotomy for surgical AVR. **(H)** Sapien 3 (left) and Perceval Sutureless (right) valves retrieved perioperatively. AVR = aortic valve replacement; CT = computed tomography; TEE = transesophageal echocardiography.

An 81-year-old female presented with New York Heart Association functional class III symptoms of breathlessness. She had undergone implantation of a 19-mm Perceval (LivaNova, Markham, Ontario, Canada) sutureless bioprosthetic valve (BPV) 2 years previously for high-risk severe native aortic stenosis. Computed tomography (CT) demonstrated stent infolding and collapse (Figure 1A). Transthoracic echocardiography confirmed degenerative BPV failure with severe valvular and paravalvular aortic regurgitation (1). Coronary angiography demonstrated unobstructed coronary arteries with fluoroscopic confirmation of valve design (Figure 1B). Following review of the imaging and clinical history, the heart team consensus was to proceed with aortic valve-in-valve transcatheter heart valve (THV) implantation using a 23-mm Sapien S3 (Edwards Lifesciences, Irvine, California) and previous fracture of the distorted bioprosthetic valve frame. The valve-in-valve procedure was performed through the transfemoral route under transesophageal echocardiographic (TEE) guidance. BPV crossing was performed using a pigtail catheter exchanged for a standard curved Safari wire (Boston Scientific, Marlborough, Massachusetts) for support. Bioprosthetic valve fracture was performed successfully using a 20-mm Atlas Gold (CR Bard, Murray Hill, New Jersey) balloon dilation catheter (Video 1). A 23-mm Sapien 3 (Edwards Lifesciences) THV was then deployed at nominal volume under rapid pacing at 210 beats/min on the Safari wire (Boston Scientific) (Figure 1C, Video 2). After THV implantation imaging demonstrated no paravalvular leakage, however, there was suggestion of a contained rupture and fistulous connection between the left ventricular outflow tract and right ventricle on both TEE (Figure 1D, Video 3) and left ventriculography (Video 4). Hemodynamic stability was maintained throughout the procedure, and the contained rupture was concluded to be benign. After THV implantation, contrast-enhanced CT (2-dimensional [2D] and 3D reconstruction) confirmed a clear contrast leak at the level of the aortic neosinuses and the waist of the THV into the right ventricular outflow tract through the valve frame (Figures 1E and 1F). Within 12 h, the patient’s hemodynamic status acutely deteriorated and required urgent resternotomy as an inpatient (Figure 1G). The THV and original Perceval sutureless valves were retrieved and the aortic valve and root replacement procedures were redone (Figure 1H). The Central Illustration depicts the transcatheter heart valve inside the Perceval bioprosthesis and the anatomical position of the fistula. Perceval valve degeneration treated with valve-in-valve THV implantation has been previously reported (1). However, this is the first report of fistula formation following valve-in-valve transcatheter aortic valve replacement in this valve, despite appropriate sizing. There are limited data, but the ENCORE (European Contained Rupture Registry) would support a benign course of initially asymptomatic contained ruptures. This contained rupture was hypothesized to have occurred following THV expansion as a result of BPV frame protrusion and was associated with late hemodynamic collapse. Further experience is required particularly when performing valve-in-valve for structural degeneration of sutureless valves, despite good outcomes in published registry data (2,3).

**Online Video 1:** Balloon valve fracture of pre-existing Perceval Sutureless valve with 20-mm Atlas Gold balloon.

**Online Video 2:** Valve-in-valve transcatheter aortic valve implant (23-mm Sapien S3), deployed under rapid pacing.

**Online Video 3:** Transesophageal echocardiography demonstrating a fistula between the left ventricular outflow tract and the right ventricle.

**Online Video 4:** Left ventriculogram with a pigtail catheter following valve-in-valve implantation, confirming contrast extravasation from the left to the right ventricle.

**Central Illustration:**
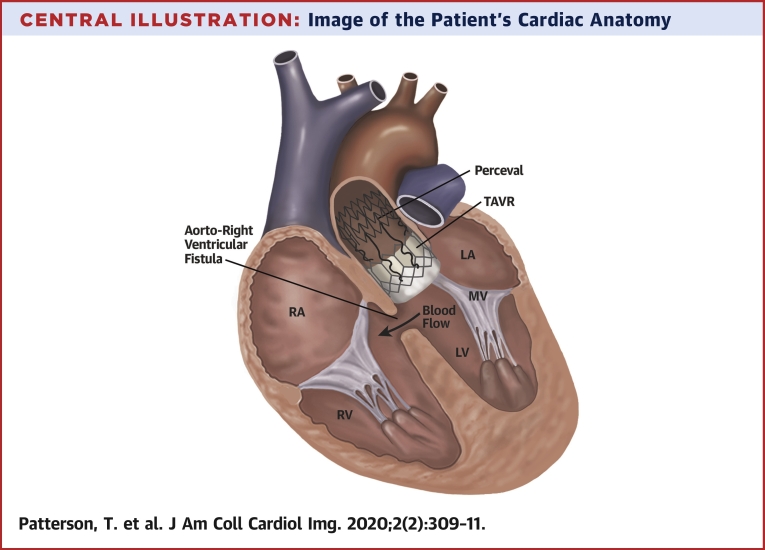
Image of the Patient's Cardiac Anatomy This demonstrates the heart in cross section, with the transcatheter heart valve (TAVR) implanted with the Perceval sutureless valve. The aorto-right ventricular fistula has created a shunt from the left ventricle (LV) to the right ventricle (RV). LA = left atrium; MV = mitral valve; RA = right atrium.
